# Hexaallylaminocyclotriphosphazene-Modified Dental Compositions for 3D Printing of Dental Crowns

**DOI:** 10.3390/polym18010053

**Published:** 2025-12-24

**Authors:** Bakary Tamboura, Pavel Yudaev, Irina Butorova, Bogdan Klyukin, Vladimir Chuev, Evgeniy Chistyakov

**Affiliations:** 1 Department of Chemical Technology of Plastics, Mendeleev University of Chemical Technology of Russia, Miusskaya sq., 9, Moscow 125047, Russia; 2 Nesmeyanov Institute of Organoelement Compounds, Russian Academy of Sciences, Vavilova St., 28, Bld. 1, Moscow 119991, Russia; 3 Belgorod National Research, Pobedy Street, 85, Belgorod 308015, Russia

**Keywords:** cyclotriphosphazene, modifier, nanoparticle, compressive strength, restorative dentistry, prosthetics

## Abstract

The development of dental restorative materials with improved physical and mechanical properties is an important area of research. In this study, hexaallylaminocyclotriphosphazene (HAP) was used to modify dental composites. HAP is a compound with multiple carbon-carbon bonds that can react with methacrylic resins to form a copolymer. HAP was synthesized by reacting allylamine with hexachlorocyclotriphosphazene and characterized it using ^1^H and ^31^P NMR spectroscopy and MALDI-TOF mass spectrometry. Molecular dynamics simulations using the MM2 force field showed that HAP has a nanosize (the diameter of a sphere eclosing the molecule is 1.3 nm), making it a suitable nanomodifier for dental composites. Using 3D printing, samples of dental methacrylic composites containing up to 10 wt. % HAP were prepared and their physicomechanical properties and antibacterial activity against gram-positive bacteria *S. mutans* were studied. As a result, it was established that the maximum flexural strength (115.1 ± 10.2 MPa) is achieved with a modifier content of 5 wt.% in the composite. The maximum value of inhibition of *S. mutans* growth in a liquid nutrient medium is achieved with a HAP content of 10 wt.% in the sample. Furthermore, with a HAP content of more than 5 wt.% in the composite, inhibition of biofilm on the material surface is observed. The resulting composite is proposed for use as dental crowns in restorative dentistry.

## 1. Introduction

The use of antimicrobial agents that act on the cell membranes of bacteria and fungi or bacterial enzymes and have an antibiofilm effect [1] is an important aspect of the therapy and prevention of infectious diseases. Antibiotics have been used as antimicrobial agents since ancient times. However, today researchers are faced with the problem of side effects of antibiotics, such as microorganism resistance to antibiotics caused by incorrectly prescribed dosage or prolonged therapy, gastrointestinal side effects (e.g., antibiotic-associated diarrhea), hematological problems, and hypersensitivity reactions [2]. Nanosized particles and antibiotic carriers [3] are acting as an alternative to commercial antibiotics that can overcome resistance.

Compared to antibiotics, metal nanoparticles can combat microbes through the simultaneous activation of various mechanisms and reduce the possibility of multiple mutations in different genes (genetic resistance factors) [4]. In turn, antibiotic carrier nanoparticles ensure long-term stability of the drug and maintain the required effective concentration [5].

In addition, nanoparticles, due to their high surface-to-volume ratio S/V and, as a consequence, high reactivity, are suitable for use in a wide range of fields of science and technology, covering not only pharmaceuticals and drug delivery [6,7], but also biomedicine [8], oil production [9], water treatment [10], food industry and agriculture [11,12], product packaging [13] and construction [14].

Nanoparticles can be used in fibrous materials intended for wound healing, for example, cellulose fiber [15], as nanofillers for synthetic rubber [16]. The addition of nanoparticles to composite materials and gel polymer systems allows the creation of materials with a unique set of properties. These include antistatic [17,18], antimicrobial [19,20], wound healing [21], anti-inflammatory [22], superhydrophobic [23] properties, high porosity [24], etc.

Nanoparticles occupy a special place in dentistry, namely in the field of therapeutic dentistry, treatment of periodontitis [25] and secondary infections, such as denture stomatitis [26], contamination of alginate dental impression material [27]. They are added to endodontic adhesives [28,29], bioceramic sealants for the treatment of periapical lesions [30], bioceramic endodontic biomaterials [31], composite resins for endocrowns [32], antimicrobial coatings for implants [33]. The use of magnetic nanoparticles, gold and silver nanoparticles, quantum dots in biosensors allows for the early detection of caries, oral cancer, and periodontal diseases [34].

However, inorganic nanoparticles (metal and metal oxide nanoparticles) can be cytotoxic to human gingival fibroblasts [35], and exhibit genotoxicity and immunotoxicity [36]. In addition, nanoparticles are capable of aggregation in the polymer matrix, which leads to the formation of large particle aggregates and deterioration of the antimicrobial and mechanical properties of the dental composite material, such as compressive and flexural strength, impact toughness [37].

To eliminate their aggregation, it is necessary to pre-treat them with surfactants that interact with surface atoms [38], graft hydrophilic polymers, such as chitosan [39], or surface chemical modification [40]. Molecular nanomodifiers that copolymerize with resins are promising alternatives to nanoparticles.

Phosphazenes, in particular organocyclophosphazenes, with a sphere diameter of less than 2 nm [41], can be classified as such molecular nanosized modifiers. The combination of inorganic (phosphazene cycle) and organic fragments (radicals at the phosphorus atom) in the structure of phosphazene modifiers, thermal stability [42], ease of functionalization at the chlorine atom of the phosphorus-chlorine bond [43,44], and their biodegradability to non-toxic and neutral ammonium phosphate [45] explains the interest of researchers in them.

Cyclophosphazenes are highly compatible with various polymers—polycarbonate [46], polypropylene [47], epoxy resins [48], gel-polymer electrolytes [49], polyurethane [50]—and can be used for sensitive components of biosensors [51] and in photoluminescent coatings for organic light-emitting diodes [52].

Cyclophosphazenes with nitrogen heterocycles in the organic radical, spirocycles, and ferrocene fragments have proven themselves to be promising non-toxic drugs for humans, possessing both cytostatic and antimicrobial action [53,54]. In particular, for amino-substituted spiro-bino-spiro-bisspirocyclotriphosphazenes, the diameters of the growth inhibition zones of bacterial and fungal strains *E. coli*, *B. cereus*, *B. subtilis*, *P. aeruginosa*, *S. aureus*, *E. faecalis*, *K. pneumoniae*, *S. typhimurium*, *E. hirae*, *P. vulgaris*, *C. albicans*, *C. krusei*, and *C. tropicalis* were in the range from 11 to 25 mm [54].

Unlike other non-toxic antimicrobial additives for dental materials, such as chalcones [55,56], which are secondary plant metabolites, modification with cyclophosphazenes allows for chemical modification of the polymer matrix of the composite material. This will achieve a stable and long-lasting antimicrobial effect.

In this paper, it is proposed to use cyclophosphazene containing nucleophilic centers (nitrogen atoms) and allyl fragments as a modifier for dental restorative materials. It is assumed that a large number of nucleophilic centers in the composition of such cyclophosphazene will lead to an imbalance in the outer membrane of bacteria and provide antimicrobial activity. In turn, the allyl fragments in the composition of cyclophosphazene will allow it to be chemically bonded with the methacrylic dental resin bis-glycidyl methacrylate (bis-GMA)/triethylene glycol dimethacrylate (TGM-3). It will prevent its release on the surface and leaching from the dental restorative material into the surrounding space under the influence of saliva, and will also improve the mechanical properties of the composite. The choice of bis-GMA/TGM-3 methacrylic resin as a resin for dental restorative material is associated with rapid curing of the resin, low polymerization shrinkage, low volatility, strength and durability of restorations based on them, good aesthetic properties, excellent polishability of the compositions, and the absence of negative consequences for human health [57,58]. In particular, in [59,60], a photocurable methacrylic resin with a zirconium dioxide/silica gel or nanosilica fillers was used to obtain a dental restorative material.

Currently, additive technologies, in particular 3D printing and CAD/CAM (Computer-Aided Design/Computer-Aided Manufacturing) technology, are becoming one of the advanced areas in the production of high-precision products in the shortest possible time [61,62]. As is known, the choice of a particular material is determined by a set of characteristics that depend on the combination of its main components. Therefore, the synthesis and study of new multifunctional oligomers with antimicrobial properties is a promising area, allowing for the production of individual products with complex geometric shapes (for example, in tissue engineering for printing dental pulp [63], for the production of implants based on polyetheretherketones with a high melting point and high melt viscosity [64]). The complex geometric shapes cannot be manufactured using traditional methods. With 3D printing, restorations are developed in gradual layers corresponding to the intended shape of the product, rather than being machined from a solid block, which contributes to material savings. In addition, 3D printing can significantly reduce the lengthy production cycle, which requires additional tooling, and also facilitate the establishment of single-piece and small-scale production of crowns. The absence of cutting tools in 3D printing allows for unrestricted movement, resulting in improved fit at the edges [65]. 3D printing also allows for more personalized and cost-effective patient care, reducing chairside time and improving the overall patient experience [66].

Due to the above advantages of 3D printing compared to traditional methods of prosthetics (using impression materials and dental models), in this work, samples for testing the dental composition were manufactured by layer-by-layer curing on a 3D printer.

## 2. Materials and Methods

### 2.1. Materials

Allylamine (≥99%), toluene (≥99%, anhydrous), triethylamine (TEA) (≥99%), diphenyl(2,4,6-trimethylbenzoyl)phosphine oxide (97%), tetrahydrofuran (anhydrous, (≥99.9%), ethanol (anhydrous, ≥99.5%), 3-hydroxypicolinic acid (98%), potassium bromide (≥99%) were purchased from Sigma-Aldrich (Saint Louis, MO, USA). Hexachlorocyclotriphosphazene (HCP, 99%) was purchased from Fushimi Pharmaceutical Co., Ltd. (Marugame, Kagawa Prefecture, Japan). Bis-glycidyl methacrylate (bis-GMA), triethylene glycol dimethacrylate (TGM-3), and silanized barium borosilicate filler with a particle size of 0.7 µm was produced by VladMiVa (Belgorod, Russia) and used without any purification. For antimicrobial activity studies, a ready-to-use nutrient medium manufactured by the Research Center for Pharmacotherapy (St. Petersburg, Russia) was used.

### 2.2. Synthesis of Hexaallylaminocyclotriphosphazene (HAP)

In a 30 mL round-bottomed flask equipped with a reflux condenser and a magnetic stirrer, 0.5 g (0.0014 mol) of HCP was dissolved in 15 mL of toluene at 110 °C. Then 0.32 g (0.0072 mol) of allylamine and 0.87 g (0.0086 mol) of triethylamine were added. The mixture was stirred for 24 h at 110 °C. The liquid phase was separated from the solid in a centrifuge, followed by decantation. Then, toluene was distilled off on a rotary evaporator, and the product was dried in a vacuum oven at 60 °C to constant weight. Yield: 0.39 g (78%).

### 2.3. Methods

To identify the obtained compounds, ^1^H and ^31^P NMR spectroscopy was used. Spectra were recorded on an Agilent/Varian Inova 400 spectrometer (Agilent Technologies, Santa Clara, CA, USA) at frequencies of 400.02 MHz and 161.94 MHz, respectively. Spectra were processed using the 1B WINNMR 5.1 (Bruker-Franzen Analytik GmbH) and MestReNova Lab-12.0.1 (Mestrelab Research) software.

The MALDI-TOF mass spectrum was recorded on a Microflex LRF mass spectrometer from Bruker Daltonik GmbH (Bruker Daltonic GmbH, Leipzig, Germany). The main features were a microScout ion source using modern pulsed ion extraction technology and a nitrogen laser with variable pulse frequency. 3-hydroxypicolinic acid was used as a matrix, and tetrahydrofuran as a solvent.

The calculation of the diameter of the sphere described around the HAP molecule was carried out using its 3D model created in the ChemBio3D program, the minimization of molecular mechanics was carried out using the MM force field.

Infrared spectra of the resulting phosphazene and acrylates mixture were recorded using KBr tablets, and the cured composition was analyzed using an ATR attachment. The spectra were recorded on a Thermo Scientific Nicolet FTIR spectrometer (Thermo Fisher Scientific, Waltham, MA, USA) with a resolution of 4 cm^−1^ in the wavenumber range from 400 to 4000 cm^−1^ at room temperature with 64 scans. The results were processed using OMNIC 9 software (Thermo Scientific).

The viscosity of the experimental samples was measured using an Atago VISCO rotational viscometer (Atago Co., Ltd., Tokyo, Japan) in accordance with ISO 2555:2018 [67]. The measurements were carried out in a dark room at a temperature of 23 °C, spindle rotation speed of 30 rpm.

The dental composition was prepared using an IKA T 25 laboratory homogenizer (IKA-Werke GmbH & Co. KG, Staufen, Germany) with subsequent vacuum degassing (10 mbar). First, acrylates bis-GMA—60% by weight and TGM-3—40% by weight were placed in the homogenizer. After their complete combination, the light-curing initiator diphenyl (2,4,6-trimethylbenzoyl) phosphine oxide 1.5% by weight and the HAP modifier in an amount of 2.5, 5, 7.5, 10% of the acrylates weight were added. A filler in an amount of 25% of the resin weight was introduced into the resulting resins and they were homogenized in a laboratory planetary mill for 60 min (speed 600 rpm).

A computer CAD model of the samples was designed in STL format and loaded into an Anycubic photon mono x 6k 3D printer (Shenzhen Anycubic Technology Co., Ltd., Shenzhen, China). Dental composites were poured into the printer’s vat to half its maximum volume. Samples were printed layer by layer: each layer of the oligomeric composite was cured with an LED source with an emission wavelength of λ_max_ = 405 nm (layer thickness—100 μm, irradiation duration—5 s). After printing, the samples were washed with ethyl alcohol and additionally kept in an LED thermal photopolymerizer (λ_max_ = 405 nm) for an hour at a temperature of 60 °C. To assess the conformity of the 3D-printed prototype with its digital counterpart, a reference model—a calibration cube (30 × 30 × 30 mm)—was selected, effectively capturing all inaccuracies. All test samples (n = 10 in each group) were printed using the same settings: layer thickness of 100 microns, model positioning relative to the print platform at 45°. After printing, the parts were washed with ethyl alcohol, and final polymerization was performed in a Form labs Form Cure LED thermal curing light (λ_max_ = 405 nm) for one hour. Digital copies were obtained using a Dentsply Sirona Ineos X5 laboratory scanner (Dentsply Sirona, Plainsboro, NJ, USA) with an accuracy limit of 2.1 µm. The obtained data was converted into an STL file containing a model description consisting of a set of points along three XYZ coordinate axes. Deviations in the actual dimensions of the geometric elements of the test pieces from the nominal values were determined by superimposing the scans using CloudCompare 2.13.2 software for processing a 3D point cloud and a triangular mesh. The open-source MeshLab system was used for editing the 3D meshes.

The morphology of the printed samples was studied using a TM3030 scanning electron microscope (Hitachi, Tokyo, Japan).

To evaluate the compressive and flexural tensile strength, specimens were printed as 4 × 6 mm cylinders and 2 × 2 × 25 mm beams, respectively. Tensile strength was measured using a model 3345 tensile testing machine (Instron, Norwood, MA, USA) with specimens loaded according to ISO 4049:2019 [68]. The crosshead speed was 0.75 ± 0.25 mm/min.

The modulus of elasticity E (MPa) was calculated using the formula:

$$E=\frac{FL^{3}}{4bh^{3}d}$$

where F is the load in the elastic deformation region of the specimen, selected on the straight-line section of the load-deformation diagram (N); d is the deformation at the selected load F (mm); L is the distance between the supports with an accuracy of 0.01 mm; b is the width of the specimen, measured immediately before the test (mm); h is the height of the specimen, measured immediately before the test (mm).

The unnotched Charpy impact strength was determined according to ISO 179:1993 [69] using a Metrotest KMM-50 pendulum impact tester. Shore D hardness was determined according to ISO 868:2003 [70].

Images of samples and their dimensions are shown in Figure S1.

To measure water absorption and water solubility, samples were prepared in the form of disks. The mass amount of water absorbed by the sample (water absorption) or leached from the sample (water solubility) was determined after 7 days of exposure to distilled water (10 mL) at a temperature of 37 ± 1 °C.

Water absorption W_sp_ and water solubility W_sl_ were calculated using the following equations:

$$W_{sp}=\frac{m_{2}-m_{3}}{v};$$

$$W_{sl}=\frac{m_{1}-m_{3}}{v};$$

where m_1_ is the conditioned mass of the specimen (μg), m_2_ is the mass of the specimen after immersion in water for 7 days (μg), m_3_ is the reconditioned mass of the specimen after immersion in water (μg), and v is the volume of the specimen (mm^3^).

Water contact angles were measured using the Goniometer LK-1 and “Drop Shape” software.

The antimicrobial activity of the samples was assessed by changing the growth activity of the test strain in a liquid nutrient medium in the presence of the test samples compared to the control variant. *Streptococcus mutans* ATTC 25,175 was used as the test strain. The prepared medium was poured into 50 mL flasks, samples and 1 mL of *Streptococcus mutans* inoculum with a cell concentration of 0.5 according to McFarland were added. Streptococcus mutans inoculum was prepared from *Streptococcus mutans* ATTC 25,175 test strain pre-grown on an agar medium (dry nutrient medium for Streptococci) for 24 h at 37 °C. The inoculated flasks were placed in a thermostat at 37 °C for 24 h. After 24 h of incubation, the growth of the strain was determined by the optical density measured on a UNICO 1201 spectrophotometer at λ = 600 nm, using cuvettes with an optical path length of 10 mm.

The resistance of dental specimens to adhesion and film formation was assessed using a method based on staining the specimens with crystal violet, followed by desorption of the dye with ethyl alcohol and spectrophotometric measurement of the resulting solution. After 24 h of incubation, the dental specimens were washed three times with phosphate buffer and stained with 0.1% crystal violet for 45 min at room temperature. The specimens were then rinsed three times with phosphate buffer to remove the dye and placed in containers with 10 mL of 95% ethyl alcohol for 45 min to desorb the dye. The resulting colored solutions were analyzed spectrophotometrically at a wavelength of λ = 630 nm. According to this evaluation method, it is assumed that the higher the optical density of the resulting dye solution, the greater the ability of microorganisms to adhere and form a film on the sample surface, and, correspondingly, the lower the resistance of the surface to microorganism adhesion and subsequent film formation.

The antimicrobial activity of the samples using the methods presented above was calculated using the formula:

$$i=\left(1-\frac{D_{mod}}{D_{N}}\right).100$$

i—degree of color/activity reduction,

$D_{mod}$—optical density of samples with modifier,

$D_{N}$—optical density of the comparison sample.

The average values of the performance characteristics of various samples were compared using one-way ANOVA followed by Tukey’s special analysis. The significance level was set up at *p* < 0.05. Five samples were used for each test.

## 3. Results and Discussion

**Figure 1:**
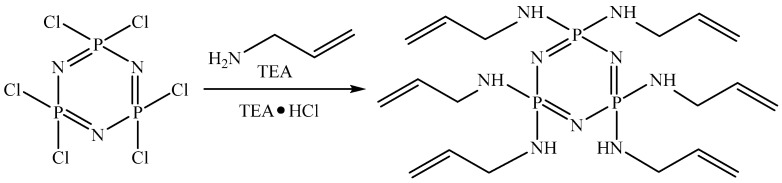
HAP synthesis scheme.

To obtain the HAP modifier, a nucleophilic substitution reaction of chlorine atoms with allyl fragments was carried out. The reaction was carried out according to the scheme shown in Figure 1.

To obtain the hexasubstituted compound, an eightfold excess of allylamine relative to HCP was used. Toluene was chosen as the solvent.

**Figure 2:**
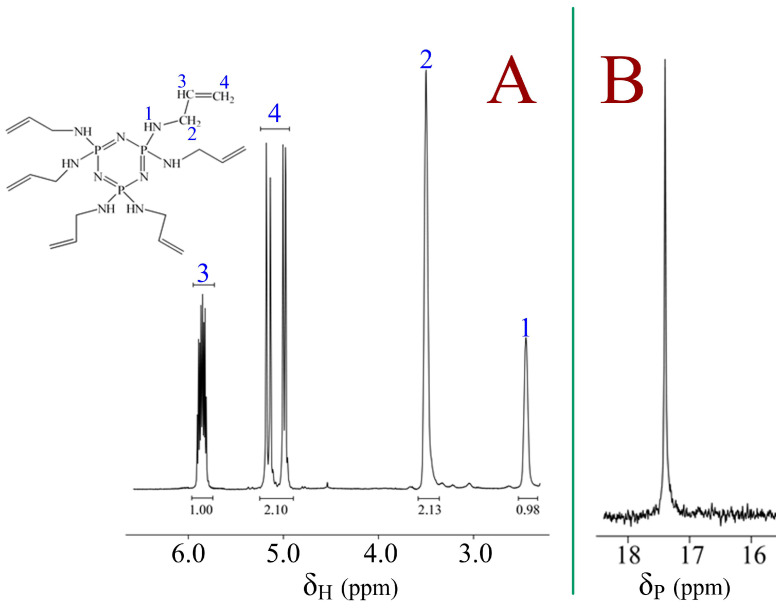
^31^P (**A**) and ^1^H (**B**) NMR spectra of HAP.

The target product was characterized by ^31^P and ^1^H NMR spectroscopy (Figure 2).

^31^P NMR spectrum of HAP (Figure 2A) shows a singlet signal at 17.13 ppm, indicating complete substitution of chlorine atoms and the absence of side reactions affecting the phosphazene cycle.

On the ^1^H NMR spectrum (Figure 2B), signals of the protons of the allyl groups are observed in the region of—5.0–6.0 ppm and the methine groups of the CH allyl fragment in the region of—3.3–3.6 ppm. This indicates the preservation of the double bond of the allyl fragment.

**Figure 3:**
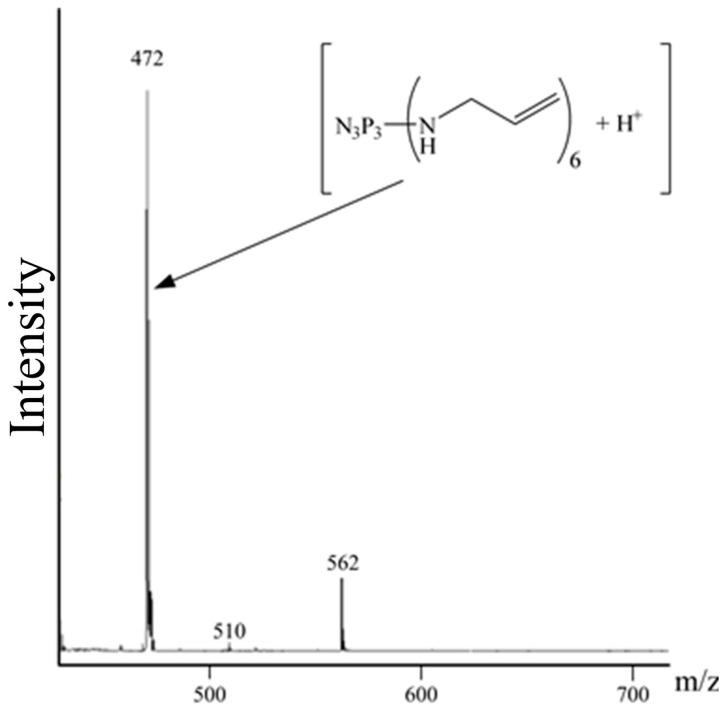
MALDI-TOF mass-spectrum of HAP.

To confirm the formation of HAP and the presence or absence of oligomeric products, MALDI-TOF mass spectrometry was performed (Figure 3).

The mass spectrum contains a molecular ion peak in the *m*/*z* 472 region, characteristic of the target compound, as well as a peak of HAP solvation by one toluene molecule with *m*/*z* 562, and a peak of the product of HAP solvation by a potassium ion in the *m*/*z* 510 region.

**Figure 4:**
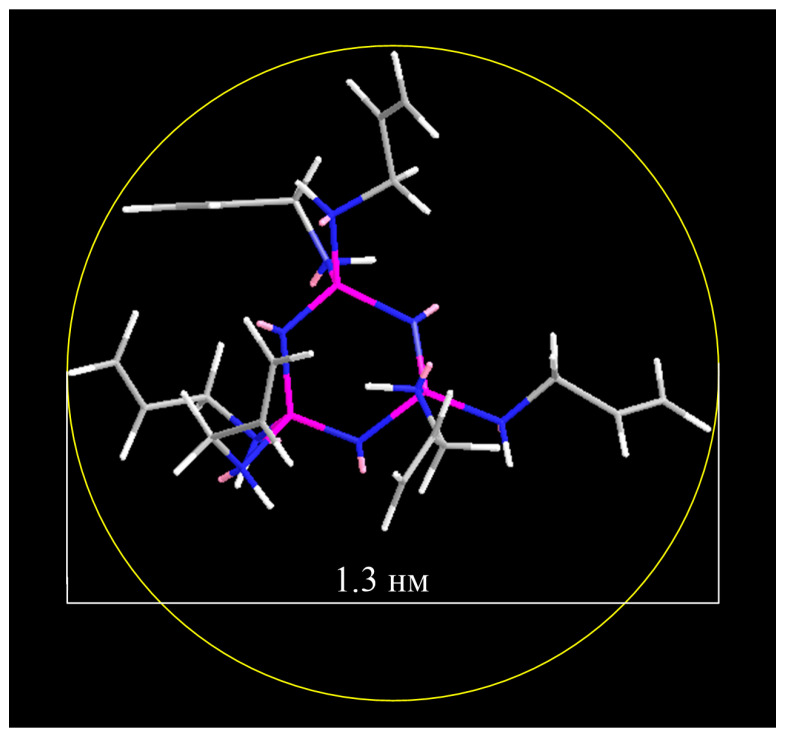
Diameter of a sphere circumscribed around a HAP molecule.

Using molecular dynamics simulation, the diameter of the sphere circumscribed around the compound molecule was calculated to be 1.3 nm (Figure 4). Nanoscale particles are known to range in size from 1 to 100 nm. Thus, HAP is a nanoscale molecule and can be hypothetically considered as a functional nanoparticle for modifying various polymeric materials, including dental ones.

**Figure 5:**
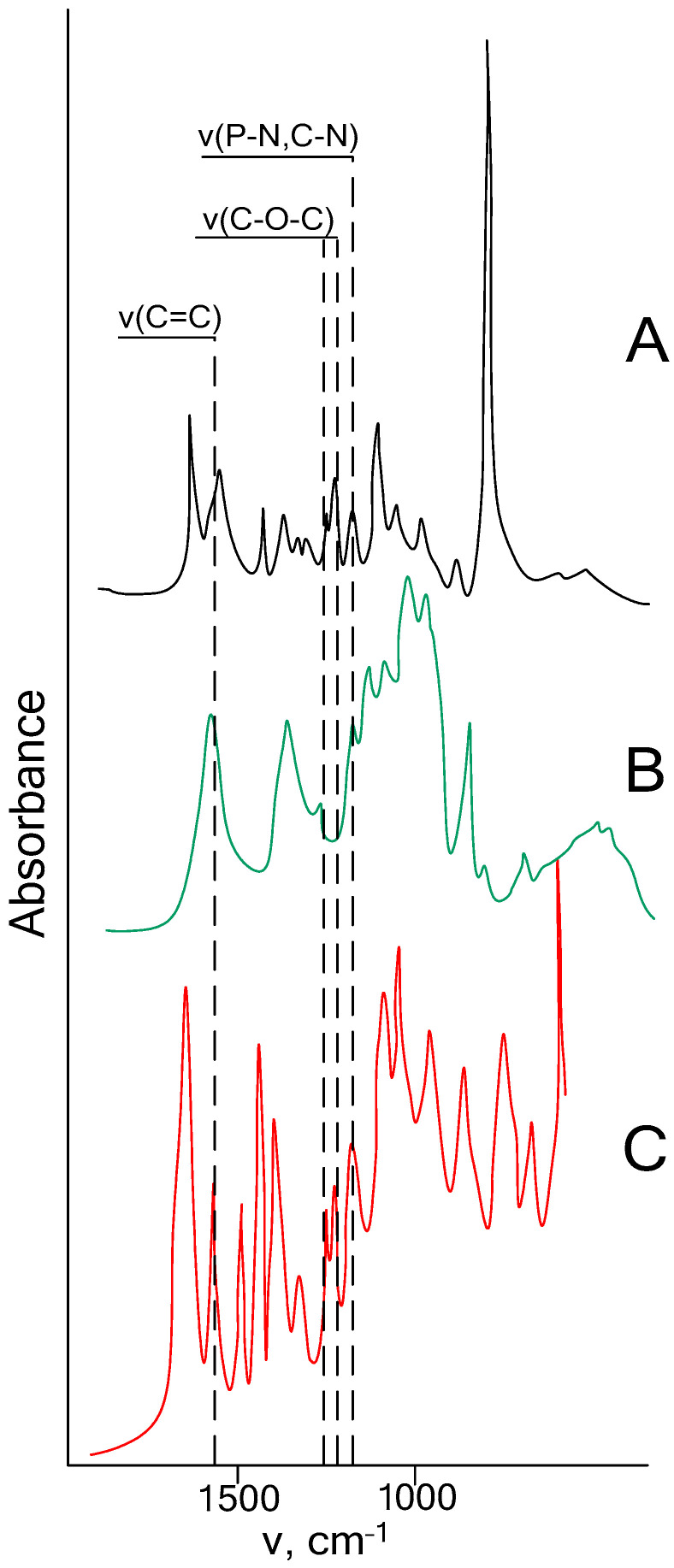
IR spectra ((**A**)—Bis-GMA/TGM-3, (**B**)—HAP, (**C**)—cured unfilled composition).

To evaluate the possibility of using HAP as a modifier for dental materials for the manufacture of crowns, unfilled dental compositions were prepared based on basic acrylic resins with the addition of various amounts of modifier—2.5, 5, 7.5, 10 wt.%. After curing, the composition with 10 wt.% was analyzed by IR spectroscopy to determine the completeness of the reaction between the resin and the modifier (Figure 5). When comparing the spectrum of HAP, the initial mixture of acrylates and the cured composition containing HAP, it can be concluded that after copolymerization, the intensity of the bands corresponding to double bonds (at 1640 cm^−1^) decreased. This indicates the involvement of allyl and methacrylate groups in the copolymerization reaction. The presence of vibration bands of cyclotriphosphazene bonds (in the region of 1200–1250 cm^−1^) confirms that the phosphazene cycle is preserved during copolymerization.

To evaluate the performance characteristics of the modified composites, they were infused with 25% by weight of silanized glass-barium particles, which are used in dental restorative materials. Cured samples were then produced from these composites using 3D printing. It should be noted that resin viscosity control is essential for successful 3D printing; it should not exceed 2500 mPa·s at 23 °C. The dynamic viscosity of the unmodified filled composition was 1100.5 ± 17.4 mPa·s. When HAP was added to the composition, the viscosity of the system increased with increasing modifier content, which is understandable given its relatively high molecular weight. However, even with a HAP content of 10 wt.%, the viscosity was 1716.7 ± 17.0 mPa·s, which is quite acceptable and ensures good printing speed.

According to scanning electron microscopy, printing produced smooth surfaces in the horizontal plane, while slight roughness was observed in the vertical plane due to the layer-by-layer application of the composite. The surface topography was dense and non-porous, with only a small amount of microimpurities on the surface, and did not change depending on the modifier content in the samples (Figure S2.1; the material texture remained unchanged after sample failure during testing; Figure S2.2). The appearance of the printed samples and the crown are shown in Figure S2.3.

The main and most important characteristic for dental crowns is the breaking stress during bending, since under chewing loads this parameter is key.

**Figure 6:**
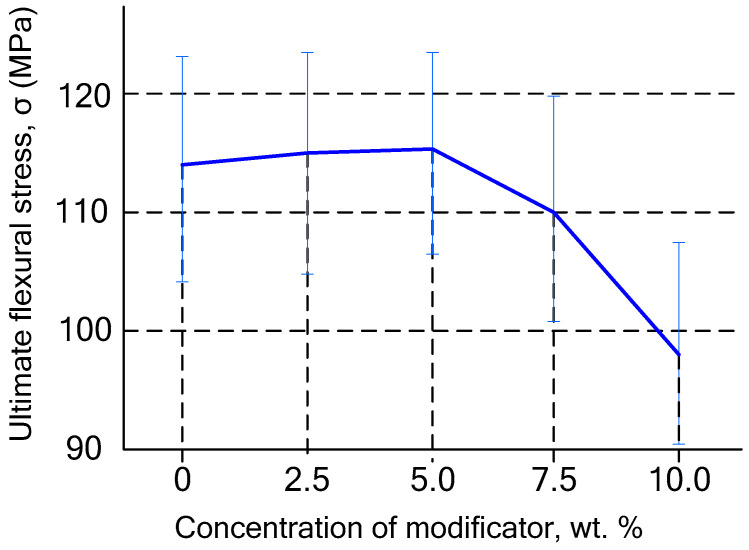
Dependence of the breaking stress in bending on the HAP content in cured filled composites (*p* > 0.05).

A study of the effect of modifier content on flexural strength revealed that a optimal HAP concentration is 5 wt.% (Figure 6), as higher modifier contents result in a decrease in strength. This can be explained by the excessively high content of allyl groups in the resin, which leads to significant chain transfer to these groups during polymerization, leading to a decrease in the density of the polymer network. Nevertheless, at all selected HAP concentrations, the compositions meet the requirements of ISO 7491-2012 [71] regarding flexural stress. According to the standard, this parameter must be at least 50 MPa.

Accordingly, further studies of the mechanical and physicochemical properties of cured filled dental composites were conducted using samples containing 5 wt.% modifier. The results of the study are presented in Table 1.

According to the obtained results, the modified compositions meet the requirements of ISO 7491-2012 [71] for dental restorative materials in terms of flexural strength, water absorption, and water solubility. According to the requirements of ISO 7491-2012, for filled compositions, the flexural strength must be at least 50 MPa, water solubility must be no more than 50 μg/mm^3^, and water absorption must be no more than 5 μg/mm^3^. It is worth noting that the addition of the modifier leads to a decrease in the values of water absorption and water solubility (*p* < 0.05). This can be explained by a decrease in the polarity of the compositions due to the aliphatic groups present in the modifier. To confirm this hypothesis, the water contact angle of composites with varying modifier contents was measured. It was found that with increasing HAP content in the samples, the contact angle increased from 30° for the unmodified composite to 41° for the composite containing 10% HAP (Figure S3).

Mechanical properties of the composites, not included in ISO 7491-2012, were investigated to further evaluate the resistance of dentures to masticatory loads (compressive stress at break), durability of the restoration (Shore hardness), resistance to fracture (impact strength), and adaptation to mechanical conditions in the body (elastic modulus).

It can be concluded that the addition of HAP did not have a negative effect on the mechanical and physicochemical properties of the composition (*p* > 0.05), in contrast to previously studied modified dental compositions [72].

The antibacterial activity of the samples of the composition containing HAP was assessed by measuring the growth activity of the test strain *S. mutans* in a liquid nutrient medium compared to the control group (nutrient medium containing *S. mutans* without the modifier). It was found that with a 2.5% HAP content in the compositions, the degree of bacterial growth inhibition was 2.9%, and with a 10% content, it reached 5.5%. These values indicate moderate activity of HAP against *S. mutans*, while the unmodified composition has no antimicrobial activity. When studying the resistance of dental samples to bacterial adhesion and film formation, it was found that compositions without a modifier and with a modifier content of up to 5% do not inhibit cell adhesion to the material. When HAP was present in samples at levels of 7.5% and 10%, the reduction in solution color intensity was 23–25%, respectively, indicating the material’s activity against pathogen biofilm formation on its surface.

## 4. Conclusions

Thus, the developed filled dental composite containing 5% HAP by weight exhibits the highest flexural strength (115.1 MPa). However, at this modifier concentration, inhibition of biofilm formation on the sample surface is not observed. Increasing the modifier content in the samples to 7.5–10% leads to a decrease in flexural strength, but inhibition of adhesion and film formation on the material surface is observed without deterioration of the physicochemical properties and other mechanical parameters.

It can be concluded that HAP has the potential to be used in dentistry as a modifier for dental crowns to prevent their colonization by pathogenic microflora. However, it is important to consider that the properties of a dental crown can be influenced by more than just the modifier amount in the material. Depending on various conditions, such as the tooth being restored, the richness and activity of the patient’s oral microflora, the pathogen strains present, the patient’s immune system, whether additional oral hygiene is performed, etc., the developed material may behave differently.

This study was aimed at demonstrating the fundamental possibility of using functional aryloxycyclophosphazenes as antimicrobial additives to dental materials and resulted in a positive outcome.

In the future, it is planned conduct additional research, including to partially change the structure of the modifier to enhance the antimicrobial effect, conduct studies not only with gram-positive but also gram-negative bacteria (for example, *P. gingivalis*), and also to study the synthesized modifier for cytotoxicity in relation to normal oral fibroblasts.

## Figures and Tables

**Table 1:** Physicochemical and mechanical characteristics of cured filled compositions.

Content HAP (wt.%)	Compressive Stress at Break, (σ_c_) MPa	Elastic Modulus (E), MPa	Shore D Hardness, Conventional Units	Water Absorption, μg/mm^3^	Water Solubility, μg/mm^3^	Charpy Impact Strength, Unnotched, kJ/m^2^
0	228.7 ± 10.8	4256.0 ± 152.0	94.5 ± 1.0	2.7 ± 0.2	1.54 ± 0.03	26.5 ± 5.0
2.5	226.6 ± 10.7 (*p* > 0.05)	4284.0 ± 152.5 (*p* > 0.05)	94.9 ± 1.0 (*p* > 0.05)	2.6 ± 0.2 (*p* < 0.05)	1.39 ± 0.03 (*p* < 0.05)	26.0 ± 5.0 (*p* > 0.05)
5	225.0 ± 10.7 (*p* > 0.05)	4345.4 ± 154.1 (*p* > 0.05)	96.0 ± 1.0 (*p* > 0.05)	1.7 ± 0.2 (*p* < 0.05)	1.07 ± 0.03 (*p* < 0.05)	25.1 ± 5.0 (*p* > 0.05)
7.5	224.0 ± 10.5 (*p* > 0.05)	4464.8 ± 153.9 (*p* > 0.05)	96.2 ± 1.0 (*p* > 0.05)	1.7 ± 0.2 (*p* < 0.05)	1.05 ± 0.03 (*p* < 0.05)	24.8 ± 5.0 (*p* > 0.05)
10	230.9 ± 10.6 (*p* > 0.05)	4505.2 ± 156.4 (*p* > 0.05)	96.4 ± 1.0 (*p* > 0.05)	1.7 ± 0.2 (*p* < 0.05)	0.95 ± 0.03 (*p* < 0.05)	26.0 ± 5.0 (*p* > 0.05)
ISO 7491- 2012 [71] requirements	-	-	-	no more 32.0	no more 5.0	-

## Data Availability

The data presented in this study are available on request from the corresponding author.

## Supplementary Materials

The following supporting information can be downloaded at: https://www.mdpi.com/article/10.3390/polym18010053/s1, Figure S1: Photos of beam and cylinder dimensions for mechanical testing; Figure S2.1: SEM images of printed samples a with different HAP content in the composite; Figure S2.2: SEM images of printed samples after mechanical properties testing (failure site); Figure S2.3: Appearance of printed samples; Figure S3: Photographs of water droplets on the surface of printed samples with different modifier contents.

## Abbreviations

The following abbreviations are used in this manuscript:

HAP	Hexaallylaminocyclotriphosphazene
NMR	Nuclear magnetic resonance
ppm	Past per million
