# Supplementary material for: Hexaallylaminocyclotriphosphazene-Modified Dental Compositions for 3D Printing of Dental Crowns
Source: Polymers (Basel). 2025 Dec 24;18(1):53. doi: 10.3390/polym18010053 (PMC12788158; doi:10.3390/polym18010053)
Supplement: Supplementary file 1 [file polymers-18-00053-s001.zip › polymers-3945148-supplementary.pdf]

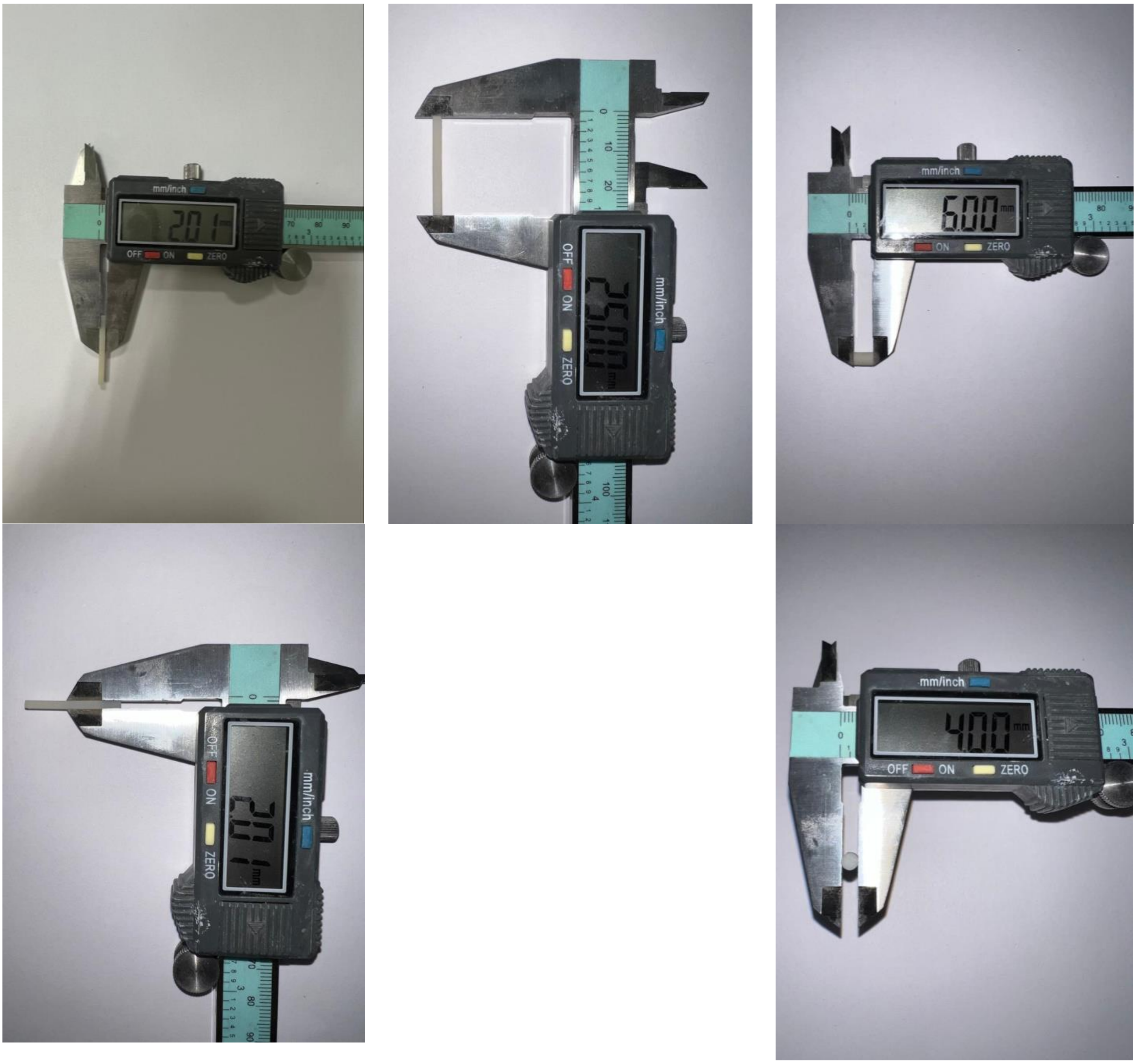

Figure S1. Photographs of the dimensions of the test specimens.

Content of HAP, wt. % **0** **5** **10**

Horizontal plane

Vertical plane

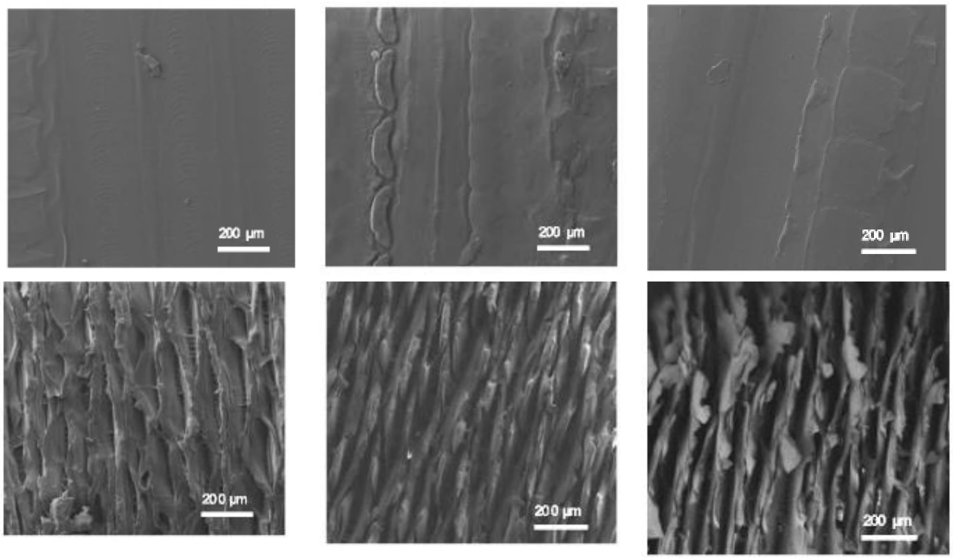

Figure S2.1. SEM images of printed samples with different HAP content in the composite.

Content of HAP, wt. %

0 5 10

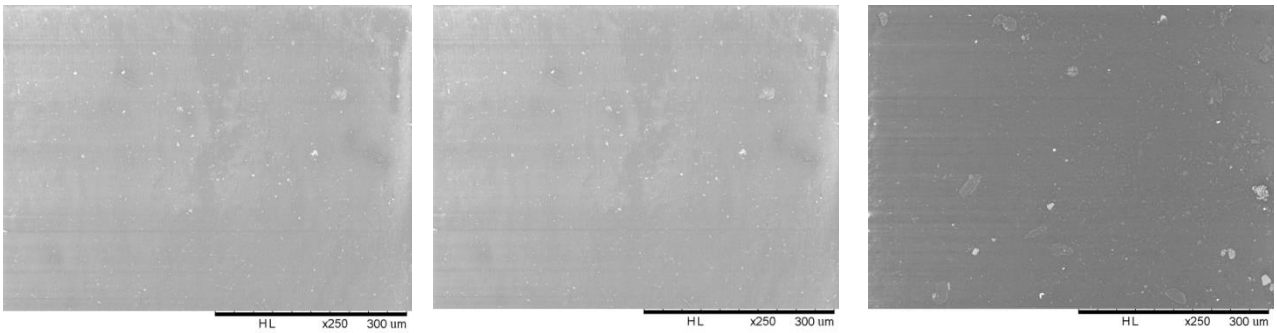

Figure S2.2. SEM photographs of printed samples after mechanical properties testing (failure site).

Samples	5 wt. % HAP, without filler	5 wt. % HAP with filler	Crown made of filled composition with 5 wt.% HAP
Appearance	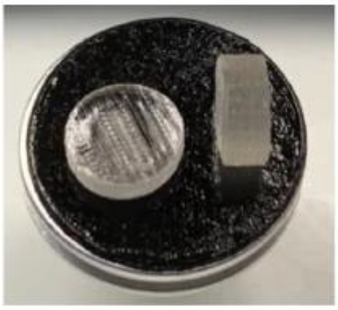	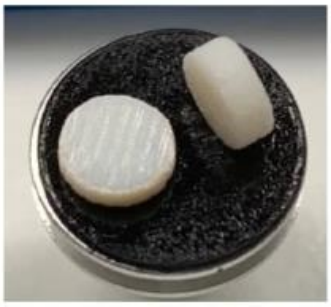	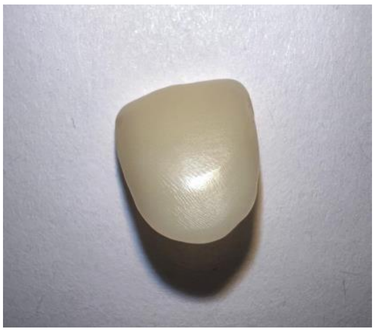

Figure S2.3. Appearance of printed samples.

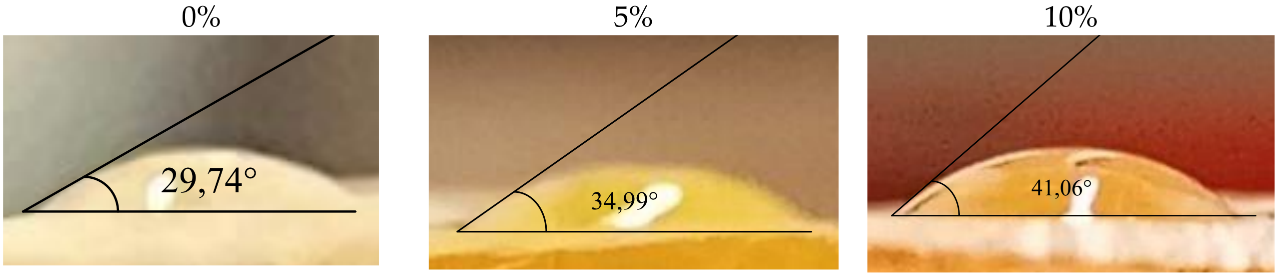

Figure S3. Photographs of water droplets on the surface of printed samples with different modifier contents.
